# Molecular Lung Imaging Following Exposure to Radiation Predicts Long-Term Survival in Rats

**DOI:** 10.3390/ijms27052485

**Published:** 2026-03-08

**Authors:** Anne V. Clough, Kathrina Mpala, Pardis Taheri, Laura Norwood Toro, Andreas M. Beyer, Tracy Gasperetti, Ming Zhao, Sarah Kerns, Heather A. Himburg, Said H. Audi

**Affiliations:** 1 Department of Mathematical and Statistical Sciences, Marquette University, Milwaukee, WI 53233, USA; anne.clough@marquette.edu; 2 Clement J. Zablocki V.A. Medical Center, Milwaukee, WI 53295, USA; 3 Department of Biomedical Engineering, Medical College of Wisconsin, Marquette University, Milwaukee, WI 53233, USA; kathrina.mpala@marquette.edu (K.M.); pardis.taheri@marquette.edu (P.T.); 4 Department of Medicine, Medical College of Wisconsin, Milwaukee, WI 53226, USA; lnorwood@mcw.edu (L.N.T.); abeyer@mcw.edu (A.M.B.); 5 Department of Physiology, Medical College of Wisconsin, Milwaukee, WI 53226, USA; 6 A.I. Virtanen Institute for Molecular Sciences, University of Eastern Finland, 70211 Kuopio, Finland; 7 Department of Radiation Oncology, Medical College of Wisconsin, Milwaukee, WI 53226, USA; tgasperetti@mcw.edu (T.G.); skerns@mcw.edu (S.K.); hhimburg@mcw.edu (H.A.H.); 8 Department of Medicine (Cardiology), Feinberg School of Medicine, Northwestern University, Chicago, IL 60611, USA; m-zhao@northwestern.edu; 9 Division of Pulmonary and Critical Care Medicine, Medical College of Wisconsin, Marquette University, Milwaukee, WI 53226, USA

**Keywords:** radiation pneumonitis, partial-body irradiation, SPECT imaging, ^99m^Tc-duramycin, ^99m^Tc-HMPAO, oxidative stress, mitochondrial DAMPs, lisinopril

## Abstract

Delayed effects of acute radiation exposure (DEARE), including radiation pneumonitis (lung-DEARE), develop weeks to months after radiation exposure. Pathway-targeted biomarkers that capture early oxidative stress and cell death could improve risk stratification and provide objective measures of mitigator efficacy. The objective was to test whether molecular lung imaging predicts long-term survival and mitigator response after irradiation. Rats received 13.5 Gy leg-out partial-body irradiation with a subset treated with the radiation-injury mitigator lisinopril. Rats underwent lung imaging at weeks 2 and 4 post-irradiation with ^99m^Tc-duramycin (cell death) and ^99m^Tc-HMPAO (oxidative stress). Plasma mitochondrial damage-associated molecular patterns (mtDAMPs) were also measured. Irradiation reduced survival with animals evidencing significant pleural effusion as an indication of radiation pneumonitis, which was mitigated with lisinopril as previously shown. Lung uptake of both imaging biomarkers increased in irradiated rats between weeks 2 and 4, consistent with worsening cell death and oxidative stress. Rats that succumbed by day 120 exhibited significantly larger increases in both biomarkers than the survivors. A predictive test was developed that predicted death by day 120 with ~70% sensitivity and specificity. Plasma mtDAMPs (ND1/2 and ATPase 6/8) increased following irradiation, and the D-loop increase from week 2 to 3 separated outcomes (increase in nonsurvivors versus decrease in survivors). Both imaging and mtDAMPs data from lisinopril-treated animals showed blunted responses. Early dual-tracer molecular lung imaging predicted long-term survival after radiation exposure and tracked mitigation with lisinopril. Circulating mtDAMPs may provide complementary systemic information to further strengthen early risk stratification after radiation exposure.

## 1. Introduction

More than 50% of survivors of accidental radiation exposure or nuclear warfare exhibit multiorgan injury [1,2,3]. A substantial proportion of those individuals will be at risk for development of Delayed Effects of Acute Radiation Exposure (DEARE), including radiation pneumonitis (lung-DEARE), typically emerging weeks to months later. No biomarker panel has yet received U.S. FDA approval for radiation mass casualty triage [3,4,5]. Also, in clinical settings, over 200,000 new lung cancer cases are diagnosed annually in the United States, with ~40% of patients undergoing radiation therapy [2,6]. Of these, up to 20% develop radiation pneumonitis even in the absence of early signs of lung injury. In both contexts, the onset and severity of injury in late-responding tissues—including the lungs, kidneys, and cardiovascular system—are highly variable and difficult to predict, with outcomes influenced by age, sex, comorbid conditions, and other yet-to-be-identified determinants [7]. Thus, readily accessible biomarkers with quick readouts, which could be utilized weeks to months following exposure, are crucial for predicting the severity of injury to late responding tissues and for tracking the efficacy of mitigators [8].

Following irradiation-evoked hydrolysis of water and direct ionization, secondary reactions result in chronic elevation of reactive oxygen species (ROS), which are formed and propagated for extended periods of time [9]. In response to increased ROS production, lung cells alter their oxidoreductive state including their glutathione content [10,11]. Moreover, increased ROS production can activate cell death processes [12]. Technetium-labeled hexamethylpropylene amine (^99m^Tc-HMPAO) has been used extensively to track changes in the activity of cellular targets that are differentially altered with oxidative stress [10,13,14,15]. Developed as a clinical brain perfusion imaging agent, it diffuses from the circulation into lung tissue where it is reduced to its hydrophilic non-diffusible form and retained within cells at a rate dependent on the cellular oxidoreductive state of the tissue, including intracellular glutathione content and mitochondrial function [10,13,16]. ^99m^Tc-duramycin is a novel SPECT marker of endothelial cell death currently in clinical trials [17,18,19]. ^99m^Tc-duramycin serves as a molecular probe that binds to phosphatidylethanolamine, which has little presence on the surface of normal viable cells but becomes exposed on the cell surface and/or accessible to the extracellular milieu with apoptosis and/or necrosis, respectively [11,14,15,18,20]. Previously, we demonstrated the utility of in vivo imaging with ^99m^Tc-HMPAO to detect oxidative stress and with ^99m^Tc-duramycin to detect endothelial cell death in rat models of lung injury [11,14,15,16,18,21,22]. We showed that increased lung uptake of ^99m^Tc-HMPAO and ^99m^Tc-duramycin correlates with advancing oxidative stress and cell death and worsening injury, whereas stable or decreasing uptake reflects protection from lung injury.

Radiation-induced oxidative stress also targets mitochondria, leading to mitochondrial DNA (mtDNA) damage, uncoupling of oxidative phosphorylation and depolarization of mitochondrial membrane potential in various organs, including lungs [23,24]. This mitochondrial injury can result in the release of mitochondrial damage-associated molecular patterns (mtDAMPs), such as damaged mitochondrial DNA (mtDNA), into the cytosol and extracellular environment. These mtDAMPS in turn can activate inflammation and exacerbate the severity of radiation-induced injury in late responding tissues, including lungs, contributing to mortality [24,25,26].

Rat models of injury associated with exposure to high doses of radiation have been developed to further understand the pathogenesis of radiation injury, identify potential targets for diagnostic/therapeutic purposes, and test novel treatments [27]. Since the radiation dose for lethal bone marrow injury is well below that for DEARE, a minimal volume of bone marrow is normally shielded in animal models to allow for the study of dose- and time-dependent assessment of DEARE and survival and to develop early biomarkers of DEARE [27,28,29,30,31]. Many such animal models have utilized female WAG/RijCmcr rats receiving partial body irradiation by shielding one limb (leg-out partial-body irradiation (PBI)) and thus sparing a minimal volume of bone marrow [27,28,29,30,31]. Fish et al. showed that following exposure to radiation doses ranging from 12.5 Gy to 14.5 Gy leg-out PBI, rats experienced a wide range of symptoms that appeared at different time points following exposure [27]. In the early acute phase (0–30 days), death occurred due to hematopoietic or gastrointestinal acute radiation syndrome. Survivors of the acute phase generally experienced a latency period of minimal morbidity. This was followed by a phase marked by DEARE to different organs (days 60–120), including lung-DEARE, with 90% of the rats that survived the acute phase succumbing by day 120 day due to radiation pneumonitis marked by the presence of pleural effusion and histological pneumonitis upon necropsy [27].

There are currently no FDA-approved countermeasures for DEARE [27,30,31]. However, pharmacologic angiotensin converting enzyme inhibitors (ACEi) are a promising class of medications, as the renin-angiotensin system regulates multiorgan response to radiation injury [27,30,31]. Lisinopril, one of the most commonly prescribed ACEi, enhances survival in irradiated rats by mitigating angiotensin II-mediated oxidative stress, inflammation, fibrosis, and apoptosis [1,30,31,32,33]. However, DEARE therapies are maximally effective if initiated during the latent period between exposure and the onset of symptoms (DEARE), which can be as late as thirty days after exposure [27,28,29,30,31]. Thus, it is critical to identify exposed patients at highest risk of lung-DEARE following either accidental exposure or radiotherapy to provide early initiation of mitigators (e.g., ACEi) to protect healthy lung tissue [34,35,36].

The objective of this study is to investigate the use of imaging with ^99m^Tc-duramycin and ^99m^Tc-HMPAO in a leg-out PBI rat model to distinguish between rats that will develop severe lung-DEARE by 120 days versus those that will recover. The animal model was also manipulated by treating subsets of rats with lisinopril to improve the likelihood of survival in order to assess the ability of the imaging protocol to monitor and predict improved outcomes.

## 2. Results

### 2.1. Survival, Body Weight, and Lung Wet/Dry Weight Ratio

**Figure 1:**
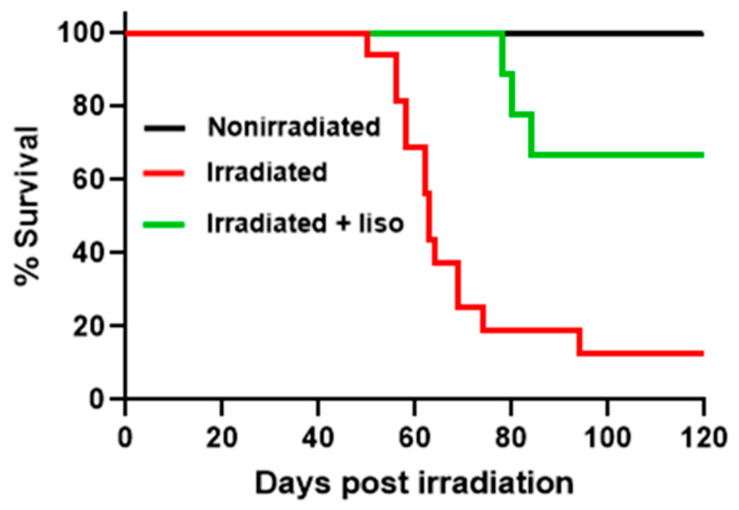
Percent of rats that survived the 120-day study period. **Black**: Nonirradiated and lisinopril-treated nonirradiated (n = 10); **Red**: Irradiated (n = 16); **Green**: Lisinopril-treated irradiated (n = 9).

In survival analyses, morbidity assessed using established IACUC criteria was used as a surrogate for mortality. The Kaplan–Meier survival curves showing the percentage of animals in all groups surviving the 120-day study period are given in Figure 1. All animals in the nonirradiated and the lisinopril-treated nonirradiated group survived for the entire length of the study (120 days). Differences between the survival curves were assessed using a logrank (Mantel–Cox) test which showed that all three survival curves are different (*p* < 0.05). Differences between the mean number of days survived were analyzed using one-way ANOVA with Tukey’s multiple comparison test and are shown in Table 1. The mean number of days survived by the irradiated animals was significantly shorter (72.2 ± 6.2 days) than the nonirradiated animals. The lisinopril-treated irradiated animals survived significantly longer (105.9 ± 7.1 days) than the irradiated animals (without lisinopril treatment), with no difference from the nonirradiated controls.

Table 1 also shows that irradiated rats had less weight gain than nonirradiated controls over the course of the study, while the lisinopril-treated rats gained weight at the same rate as the nonirradiated controls. At the time of euthanasia, there was a significant amount of pleural effusion in the chest cavity of the irradiated animals (6.5 ± 0.9 g); this was reduced significantly in the lisinopril-treated irradiated animals (1.1 ± 1.1 g). As shown in Table 1, the lung wet/dry weight ratio of irradiated rats tended to be higher than nonirradiated controls but did not reach significance. There was no significant difference between lisinopril-treated irradiated and lisinopril-treated nonirradiated rats.

### 2.2. Lung Imaging

**Figure 2:**
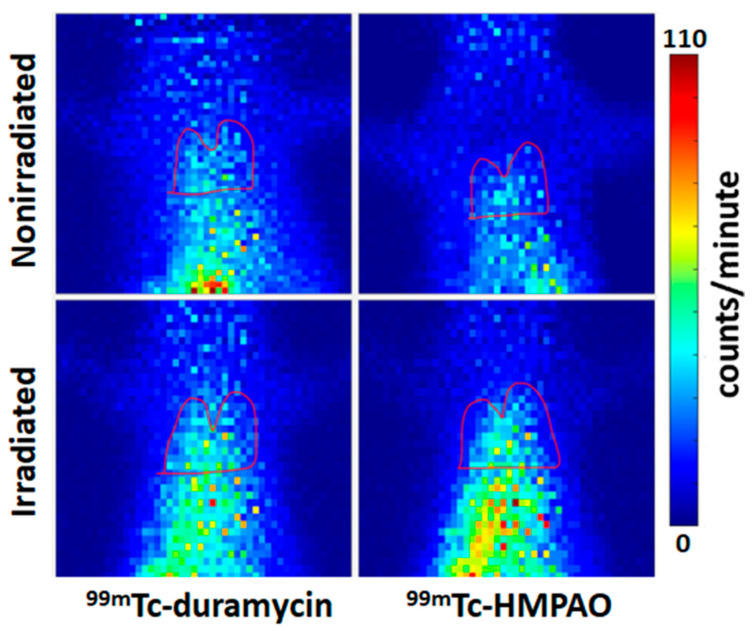
Planar images of ^99m^Tc-duramycin (^99m^Tc-DU, **left**) and ^99m^Tc-HMPAO (**right**) lung uptake in nonirradiated (control, **top**) and irradiated (week 4 post-irradiation, **bottom**) rats. Lung boundaries were identified by superimposing the ^99m^Tc-MAA image that shows the lung ROI. Horizontal lines were drawn at the midpoint of the lungs to avoid any liver contribution.

Figure 2 shows representative examples of average ^99m^Tc-duramycin (left) and ^99m^Tc-HMPAO (right) images. The nonirradiated animals (top) show less biomarker uptake in the red lung ROI than the irradiated (week 4 post-irradiation) animals (bottom) for both ^99m^Tc-duramycin (left) and ^99m^Tc-HMPAO.

**Figure 3:**
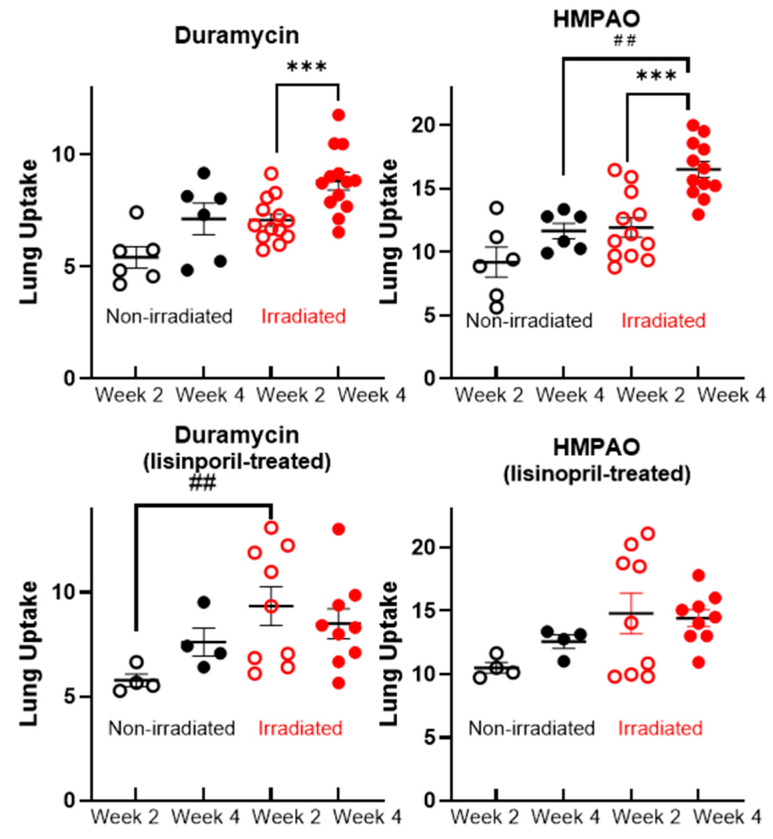
**Top**: Lung uptake of ^99m^Tc-duramycin (**left**) and ^99m^Tc-HMPAO (**right**) in nonirradiated control (black symbols) and irradiated (red symbols) rats at week 2 and week 4 following irradiation. Open circles for week 2 and closed circles for week 4 post irradiation. *** Irradiated week 4 different from irradiated week 2 (paired *t*-test, *p* < 0.001). ^##^ Irradiated different from nonirradiated in week 4 HMPAO uptake (unpaired *t*-test *p* < 0.01). **Bottom**: Lung uptake of ^99m^Tc-duramycin and ^99m^Tc-HMPAO in lisinopril-treated nonirradiated control and irradiated rats at week 2 and week 4 following irradiation. ^##^ Irradiated different from nonirradiated in week 2 ^99m^Tc-duramycin (unpaired *t*-test, *p* < 0.01).

Quantification of lung uptake of ^99m^Tc-duramycin (left) and ^99m^Tc-HMPAO (right) at weeks 2 and 4 is shown in Figure 3. The top panels show that for both biomarkers there was significantly more uptake at week 4 than week 2 in the irradiated animals (^99m^Tc-duramycin: 8.82 ± 0.40 versus 7.08 ± 0.27; ^99m^Tc-HMPAO: 16.51 ± 0.64 versus 11.93 ± 0.76). No similar increase occurred in the nonirradiated controls. Moreover, there was significantly more ^99m^Tc-HMPAO uptake at week 4 in the irradiated animals than the nonirradiated controls (16.51 ± 0.64 versus 11.66 ± 0.61).

Results for the lisinopril-treated rats are shown in the bottom panels of Figure 3. In these animals, no significant increase in either ^99m^Tc-duramycin or ^99m^Tc-HMPAO lung uptake between week 2 and week 4 was observed. There was a significant difference in ^99m^Tc-duramycin lung uptake between the nonirradiated control (5.78 ± 0.30) and the irradiated (9.34 ± 0.93) animals at week 2, although this difference was not observed at week 4. For ^99m^Tc-HMPAO, there was no significant difference in lung uptake between the nonirradiated controls and irradiated animals at either week 2 or week 4.

**Figure 4:**
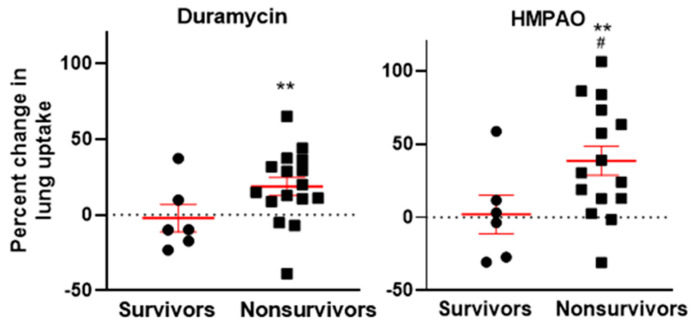
Percent change in lung uptake between week 2 and week 4 post-irradiation of ^99m^Tc-duramycin (**left**) and ^99m^Tc-HMPAO (**right**). Rats are grouped into those that survived for 120 days (survivors; n = 1 irradiated and n = 5 irradiated with lisinopril treatment) versus those that succumbed prior to 120 days (nonsurvivors; n = 13 irradiated and n = 3 irradiated with lisinopril treatment). Group mean is represented by the solid horizontal bar for each group. ** Percent change in lung uptake different from 0, (one-sample *t*-test, *p* < 0.01). ^#^ Nonsurvivors different from survivors (HMPAO) (unpaired *t*-test. *p* < 0.05).

The data were segregated based on those animals that survived for the 120-day study period (survivors) versus those that succumbed prior to the study end (nonsurvivors). Figure 4 shows the percent change in lung uptake of both ^99m^Tc-duramycin (left) and ^99m^Tc-HMPAO (right) in the survivor and nonsurvivor groups. For ^99m^Tc-duramycin, a one-sample *t*-test shows that uptake increased significantly (18.5 ± 0.6%) between week 2 and week 4 in the nonsurvivors but not the survivors. For ^99m^Tc-HMPAO, there was also a significant increase in the nonsurvivors (38.5 ± 1.9%) but not the survivors. Figure 4 (right) also shows that for ^99m^Tc-HMPAO, the mean increase in uptake was significantly higher in the nonsurvivors than the survivors (unpaired *t*-test).

### 2.3. Plasma Levels of mtDAMPs

**Figure 5:**
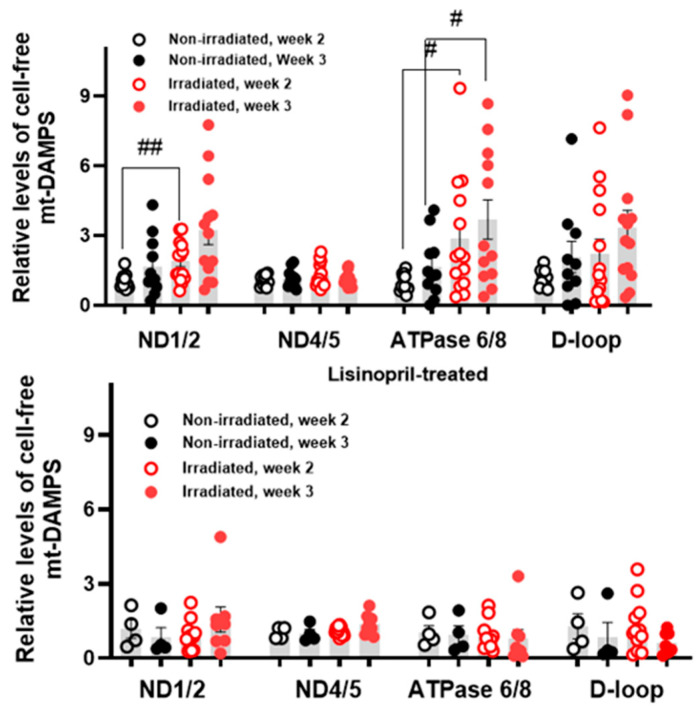
Plasma levels of mtDAMPs at weeks 2 and 3 following irradiation. Values represent fold-change in gene expression relative to the mean week 2 value from the corresponding nonirradiated group: nonirradiated controls (**top**) or nonirradiated lisinopril-treated (**bottom**). ^##^ Irradiated different from nonirradiated (unpaired *t*-test, *p* < 0.01). ^#^ Irradiated different from nonirradiated (unpaired *t*-test, *p* < 0.05).

Figure 5 shows plasma mtDAMP levels normalized to nonirradiated control levels. ND1/2 and ATPase 6/8 at week 2 following irradiation were both significantly elevated (~1.8 and ~2.9 fold, respectively) compared to nonirradiated controls, with a further increase (~3.7-fold) in ATPase 6/8 at week 3 post-irradiation. In contrast, no irradiation-associated differences were observed in lisinopril-treated rats.

**Figure 6:**
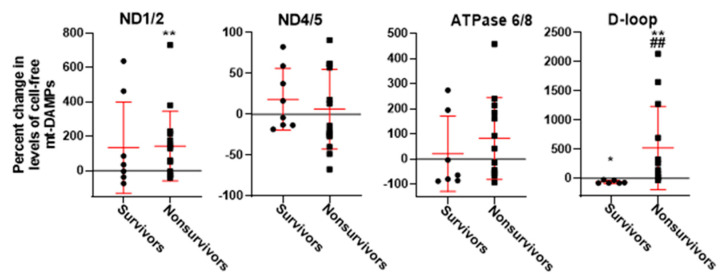
Percent change in plasma levels of mtDAMPs between week 2 and week 3 post-irradiation. Rats are grouped into those that survived for 120 days (survivors) versus those that succumbed prior to 120 days (nonsurvivors). Percent change in lung uptake is different from 0 (one-sample *t*-test, ** *p* < 0.01 or * *p* < 0.05). ^##^ Nonsurvivors different from survivors (unpaired *t*-test, *p* < 0.01).

The mtDAMPs data from the irradiated rats were segregated into survivors versus nonsurvivors as was done for the imaging data. Figure 6 depicts the percent change in fold change between week 2 and week 3 for each mtDAMP. ND1/2 levels increased significantly from week 2 to week 3 following irradiation in nonsurvivors (43%, *p* = 0.008) but not in survivors. For D-loop, nonsurvivors had a significant increase (422%, *p* = 0.005), whereas survivors had a significant decrease (−61%, *p* = 0.031). Consistent with this, the mean D-loop percent increase was significantly greater in nonsurvivors than survivors (*p* = 0.002).

### 2.4. Statistical Prediction Model

**Figure 7:**
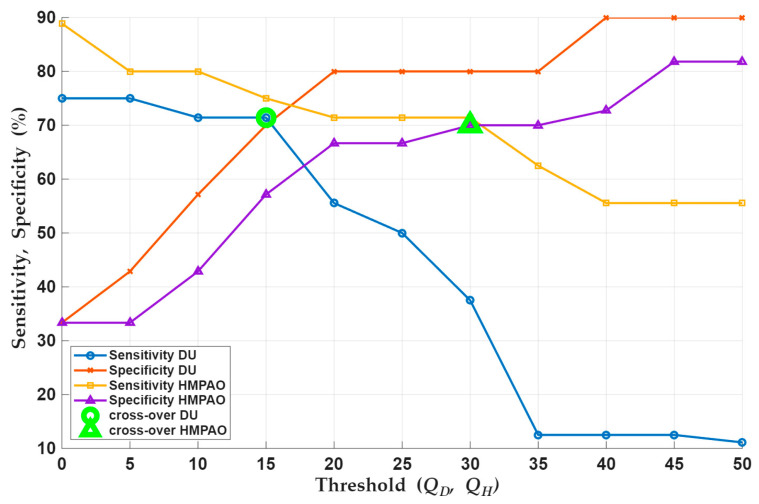
Sensitivity (blue) and specificity (red) for ^99m^Tc-duramycin and sensitivity (yellow) and specificity (purple) for ^99m^Tc-HMPAO as a function of different threshold values. Green symbols indicate crossing points of sensitivity and specificity curves for each biomarker.

We investigated the ability of lung uptake of ^99m^Tc-duramycin and ^99m^Tc-HMPAO to predict long-term survival following radiation exposure in these animals. The objective was to determine the percent increase in ^99m^Tc-duramycin and ^99m^Tc-HMPAO lung uptake between week 2 and week 4 (threshold defined as *Q*) that would be optimally predictive of long-term survival, given the limited data set. To begin we classified each rat using the lung uptake from imaging data and long-term survival as shown in Table 2:

To determine the optimal threshold, *Q*, for each imaging biomarker, a computational loop iterated through a range of threshold values, each time determining the resulting sensitivity [TP/(TP + FN)] and specificity [TN/(TN + FP)] using the data from each animal. Figure 7 shows a graph of the resulting sensitivity and specificity as a function of the threshold for ^99m^Tc-duramycin and ^99m^Tc-HMPAO. As expected for both ^99m^Tc-duramycin and ^99m^Tc-HMPAO, the sensitivity and specificity curves are inversely related. Based on these plots, we selected a threshold *Q_D_* for ^99m^Tc-duramycin and *Q_H_* for ^99m^Tc-HMPAO where the corresponding sensitivity and specificity curves intersect, i.e., *Q_D_* = 15% and *Q_H_* = 30%. We then reanalyzed the data to examine the predictive power of combining the two imaging agents by reclassifying each rat according to the definitions in Table 3.

Thus, an individual rat was deemed to have a “positive test” result, i.e., prediction of death by 120 days, if the change in its lung uptake of both ^99m^Tc-duramycin and ^99m^Tc-HMPAO was greater than 15% and 30%, respectively. This combined prediction test resulted in an overall sensitivity of 71% and specificity of 70%.

## 3. Discussion

Using a PBI rat model, we developed and evaluated a robust imaging protocol using previously investigated SPECT biomarkers of lung injury to distinguish subjects with radiation exposure destined for severe radiation pneumonitis and death from those likely to recover without treatment. The rat model was manipulated by treating subgroups of rats with lisinopril, a radiation injury mitigator. The protocol involved imaging each animal at two and four weeks post-irradiation with both ^99m^Tc-duramycin and ^99m^Tc-HMPAO and using the percent change in lung uptake of each biomarker as a predictor of survival (or death). The key findings were as follows: (i) irradiated rats showed progressive increases in lung uptake of ^99m^Tc-duramycin and ^99m^Tc-HMPAO, consistent with progressive radiation-induced cell death and oxidative stress; (ii) nonsurvivors exhibited significantly greater increases in both imaging biomarkers than survivors; (iii) ^99m^Tc-duramycin and ^99m^Tc-HMPAO lung uptake in lisinopril-treated rats tracked the reduction in lung injury and improvement in survival observed in this group; (iv) plasma levels of two mtDAMPs increased following irradiation, and lisinopril treatment blunted these irradiation-associated increases; and (v) using the percent change in lung uptake of each imaging biomarker as a predictor of survival (or death), a predictive “test” with 71% sensitivity and 70% specificity was developed, based only the relatively small sample size in this study.

It has previously been established that the ACE inhibitor lisinopril improves survival in this model [1,30,32,33]. As such, in the present study it was used to modulate the severity and outcome distribution within the animal cohorts, thereby broadening the range of phenotypes evaluated, rather than to establish efficacy. This provided a means for determining whether our imaging strategy could detect a therapeutic response by reliably predict survival in irradiated, lisinopril-treated rats with higher survival rates.

For nonirradiated and lisinopril-treated nonirradiated rats, there was increasing body weight and stable (unchanging) biomarker uptake between weeks 2 and 4 post-irradiation (Table 1, Figure 3). This increase in body weight per day for those rats was consistent with previously reported weight gain for nonirradiated rats [37]. This suggests that the ^99m^Tc-duramycin and ^99m^Tc-HMPAO imaging protocol had no observable significant effect on the overall health of the animals.

Radiation pneumonitis was quantified by pleural effusion weight, which averaged 6.5 g in irradiated rats compared with 0 g in nonirradiated rats (Table 1). Consistent with its survival benefit (Figure 1), lisinopril also mitigated pneumonitis, as reflected by a significant decrease in pleural effusion (1.1 g, Table 1).

There is ample evidence that radiation-induced lung injury is initiated by radiation-induced increases in ROS production and cellular damage, followed by sustained oxidative stress, inflammation, endothelial and epithelial cell injury, and vascular permeability changes [21,23,24,27]. As such, oxidative stress and cell death play key roles in the progression of radiation-induced lung injury. In this context, ^99m^Tc-HMPAO provides a functional measure of tissue cellular oxidoreductive state including intracellular glutathione content and mitochondrial function [10,13,16]. In parallel, ^99m^Tc-duramycin provides a functional measure of endothelial cell death [17,18,19,38].

Irradiated rats showed significantly more lung uptake of ^99m^Tc-duramycin and ^99m^Tc-HMPAO at week 4 than nonirradiated rats (Figure 3). Furthermore, irradiated rats showed a significant increase in the uptake of both biomarkers between week 2 and week 4 after irradiation, as an indication that these biomarkers are tracking the progressive lung injury associated with radiation pneumonitis. In irradiated rats that survived for 120 days, the results show that there was no significant difference in lung uptake during weeks 2 to 4 (Figure 4). However, nonsurviving rats had a significant increase in ^99m^Tc-duramycin and ^99m^Tc-HMPAO lung uptake, indicative of increased cell death and oxidative stress, respectively, in these nonsurvivors.

The ACE inhibitor lisinopril has been shown to be a potent mitigator of radiation pneumonitis [1,30,31,32,33]. Thus, it was administered to a subset of rats as a means of manipulating the animal model to increase survival in irradiated rats. The survival curves in Figure 1 show that lisinopril improved survival in the radiated rats from ~15% to ~70%. Previous studies have documented that lisinopril-treated rats often exhibit clinical signs of radiation pneumonitis such as weight loss and labored breathing, particularly in the first weeks following irradiation [28]. Consistent with induction of injury, an increase in ^99m^Tc-duramycin lung uptake is observed at 2 weeks in the lisinopril-treated animals. However, further increases in ^99m^Tc-duramycin, and ^99m^Tc-HMPAO, lung uptake from week 2 to week 4 are suppressed (Figure 3) as lisinopril mitigation prevents lethal injury progression, demonstrating its ability to mitigate radiation pneumonitis and improve survival in this study. Figure 3 also shows a significant difference between ^99m^Tc-duramycin lung uptake in nonirradiated versus irradiated rats at week 2 in the lisinopril-treated rats, but not in the untreated rats. One possible explanation for this result is that at week 2, even the survivors have evidence of lung injury, which subsequently begins to resolve by week 4. This is the motivation for subsequently segregating the data into the survivors and nonsurvivors as done in Figure 4.

A simple model was developed to predict survival to 120 days. The optimal predictor was selected as the percent increase in lung uptake of ^99m^Tc-duramycin and ^99m^Tc-HMPAO that corresponded to the intersection of the computed sensitivity and specificity curves, given the inherent trade-off between these characteristics. With our limited data, the model predicts, with ~70% sensitivity and specificity, that rats that exhibit a 15% increase in ^99m^Tc-duramycin and a 30% increase in ^99m^Tc-HMPAO lung uptake between week 2 and week 4 will survive for 120 days (Figure 7). These results were obtained with relatively small group sizes; increasing *n* would likely improve both sensitivity and specificity and permit the use of separate model-training versus validation data sets. Also, it may be that with increased group sizes, ^99m^Tc-HMPAO imaging alone would be sufficient to use for prediction. We could also consider increasing the number of imaging time points as means of improving the model, although this may be burdensome to translate to humans. Finally, we note that given the relatively low-risks associated with use of lisinopril and the high-risks associated with failing to identify an individual with significant radiation exposure, a predictive test that favors higher sensitivity at the expense of some specificity could be considered.

The imaging protocol presented here involves administration of radioactive biomarkers. Their use is proposed at trace doses, unlikely to result in any additional injury to the subject, as demonstrated by many years of widely accepted nuclear, SPECT, and PET imaging. Nonetheless, this study demonstrated the potential for oxidative stress (^99m^Tc-HMPAO) and cell death ^99m^Tc-duramycin) biomarkers to predict survival/death; other noninvasive in vivo imaging modalities such as ultrasound or MRI that can track these pathways could also be considered/evaluated.

Circulating mtDAMPs, including cell-free mtDNA fragments, represent a complementary systemic biomarker that may reflect mitochondrial injury and inflammatory signaling. Lui et al. have highlighted the potential value of circulating indicators for earlier detection and risk modeling, especially when integrated with functional measures [39]. As such, in the present study we observed irradiation-associated increases in plasma levels of particular mtDAMPs (ND1/2 and ATPase 6/8, Figure 5) and a differential survival-associated percent change in plasma level of D-loop between weeks 2 and 3 post-irradiation, with marked increases in nonsurvivors and decreases in survivors (Figure 6). Moreover, our results show that lisinopril attenuated these irradiation-associated mtDAMPs signatures (Figure 5), suggesting that lisinopril mitigated systemic injury signals as well as lung injury endpoints. Integrating mtDAMPs data with lung imaging data may improve the performance (i.e., increase sensitivity and specificity) of the statistical prediction model for early survival stratification following irradiation.

Our results in this rat model suggest that further investigation is warranted. Future work should test the robustness of the findings at different radiation doses, in both males and females, in different age populations, in additional exposure scenarios, and importantly, in humans [27,30,31,32]. Previous studies have demonstrated biological sex differences in both radiation sensitivity and the pharmacokinetics/pharmacodynamics of ACE inhibitors (ACEi) [27,31]. For instance, for the 13.5 Gy leg-out partial-body irradiation (PBI) model used in this study, 120-day mortality is significantly higher in males than in females [27]. Although ACEi improve survival and reduce lung DEARE in both male and female rats [1,30,32,33], the efficacy of lisinopril in protecting against radiation-induced injury has been shown to be sex-specific, with mortality significantly higher in male than female rats [31].

While planar imaging is well-suited for high-throughput studies, it may underestimate spatial heterogeneity of lung injury. Thus, SPECT/CT could improve regional localization and specificity. Also, the above predictive thresholds for change in lung HMPAO and duramycin should be validated in an independent cohort, and development of survival prediction rules using a classification-tree (CART) approach based on both imaging and mtDAMPs data may further strengthen survival prediction and perhaps enable early risk stratification [22].

Our study extends results obtained previously by Medhora et al. in which SPECT imaging with the pulmonary perfusion marker ^99m^Tc-MAA (macroaggregated albumin) was combined with measures of circulating microRNA and white blood cell counts in plasma to predict survival [22]. Our approach is distinct from that work in that the SPECT biomarkers were selected to be pathway-specific (cell death and oxidative stress), with the long-term clinical goal of predicting development of radiation pneumonitis but also as a research tool for investigation of the cellular mechanisms involved in radiation-induced lung injury and assessment of mitigators. Other minimally invasive imaging modalities have been proposed for detecting and monitoring early signs of radiation pneumonitis include CT [40,41], MRI [42], and PET [43]. Again, much of this work is focused on structural or other nonspecific changes that occur long after the 2–4-week latency period chosen in our study. Early identification of injury is critical for effective mitigation.

## 4. Materials and Methods

### 4.1. Materials

HMPAO (Ceretec^®^) was purchased in kit form from GE Healthcare (Arlington Heights, IL, USA), and technetium-labeled macroaggregated albumin (^99m^Tc-MAA, particle sizes 20–40 µm) was purchased from Cardinal Health (Wauwatosa, WI, USA). Duramycin (3035 g/mole MW) kits were prepared as previously described [15,18]. Primer sets for mtDAMPs qRT-PCR analysis were purchased from Integrated DNA Technologies (Coralville, IA, USA).

### 4.2. Radiation Exposure and Treatment

All treatment protocols were approved by the Institutional Animal Care and Use Committees of the Clement J. Zablocki Veterans Administration Medical Center, Medical College of Wisconsin (MCW), and Marquette University (MU).

WAG/RijCmcr female rats of Wistar origin (11–12 weeks old, 158.9 ± 1.32 (SEM) g, n = 38) were bred and housed at MCW. Animals were acclimated for 3 days prior to experimental procedures and housed in standard cages (three rats per cage) under controlled environmental conditions (22 ± 2 °C; 30–60% relative humidity) with a 12:12 h light–dark cycle.

Irradiation was performed on non-anesthetized rats immobilized in a plastic jig with one leg externalized and shielded with lead such that the approximate bone marrow shielding was 5–8% [27]. The rats were irradiated (leg-out PBI) using the Precision XRAD320 orthovoltage X-ray system (Kub Technologies, Inc, Stratford, CT, USA) (320 kVp, 12.5 mA, 1.65 mm Cu half value layer, filtration with 0.135 mm Cu and 2 mm Al) with a dose of 13.5 Gy (leg-out PBI) at a rate of 165.6 cGy/min. Radiation exposures were verified to be within 5% of the target dose by the Radiation Nuclear Countermeasure Program (RNCP) Irradiator Dosimetry Program (Dr. Larry DeWerd, Radiobiology Dosimetry Program, University Wisconsin-Madison). Randomization was used to allocate animals to control and irradiation groups. Specifically, rats designated for each cohort were randomized prior to irradiation using simple randomization. Following randomization, animals were ear-punched for identification prior to the beginning of the study. Group assignments were implemented by a technician not involved in outcome assessment, helping to minimize allocation and assessment bias. This study consisted of five cohorts of rats: three without and two with lisinopril treatment. Each cohort consisted of two nonirradiated and six irradiated rats. Female WAG/RijCmcr rats were used because the established leg-out PBI model has been most extensively characterized in female rats of this strain, enabling direct comparison with prior work and reported injury endpoints [27,28,29,30,31,44,45,46].

All rats were fed Tekald 2018 rodent chow (Inotiv, West Lafayette, IN, USA) and given supportive care including powdered food, hydration, and the antibiotic enrofloxacin (10 mg/kg/day, days 2–14 post irradiation) [28]. An additional group of rats was administered lisinopril (4 mg/kg/day) via drinking water starting at day 7 following irradiation [28,30,31]. Every four days, the rats were weighed and monitored for euthanasia criteria. For survival endpoints, morbidity, defined according to established IACUC criteria, was employed as a surrogate measure of mortality. This study consisted of four groups of rats: controls with no irradiation (n = 6), irradiated (n = 16), lisinopril-treated with no irradiation (n = 4), and lisinopril-treated with irradiation (n = 9). Plasma samples were collected from all animals at both day 13 (week 2) and day 21 (week 3) post-irradiation to minimize stress on the animals. All animals were imaged at both day 14 (week 2) and day 28 (week 4) post-irradiation, after which the rats were rehoused and monitored for euthanasia criteria or until day 120 post-irradiation.

### 4.3. Plasma Levels of mtDAMPs

For blood collection, rats were anesthetized with isoflurane (5%). A 23 G needle was then inserted into the center of the jugular fossa to access the jugular vein. No more than 1.5 mL of blood (max of 3 mL over 3 weeks) was withdrawn into the syringe coated with EDTA. EDTA was added to achieve 50 µL per 1 mL of blood collected. Samples were then centrifuged at 1000× *g* for 10 min at 4 °C, after which plasma was carefully removed without disturbing the buffy coat, aliquoted, and stored at −80 °C until use for analysis of circulating mtDAMP levels. Specifically, gene expression of cell-free mitochondrial DNA (mtDNA) fragments ATP synthetase subunits 6 and 8 (ATPase 6/8), NADH dehydrogenase subunits 1 and 2 (ND1/2), NADH dehydrogenase subunits 4 and 5 (ND4/5), and displacement loop (D-loop) was quantified using quantitative real-time polymerase chain reaction (qPCR) [47,48,49,50]. Briefly, plasma was diluted 1:40 in nuclease-free water and heat-treated at 95 °C for 30 min. Primer sets for qRT-PCR analyses targeting four regions of the rat mtDNA in plasma are listed in Table S1 in Supplemental Materials (Integrated DNA Technologies, Coralville, IA, USA) [51]. For each primer set, 1 µL of diluted, heat-treated plasma was added per well, and reactions were run using the Qiagen QuantiNova SYBR Green RT-PCR Kit (QIAGEN, LLC, Germantown, MD, USA). Relative levels were calculated using the ΔC_T_ method [49,52], comparing samples from irradiated rats with those from control nonirradiated rats.

### 4.4. Imaging Studies

In vivo imaging studies described below were conducted on all rats from each group. The number of rats for each group was chosen to achieve a power ≥ 85% using power analysis (ANOVA power) based on previously published means and standard deviations of the lung uptake of ^99m^Tc-HMPAO and ^99m^Tc-duramycin [10,13,14,15,16,18]. In all imaging studies at least one control rat was imaged using the same prepared batch of radiopharmaceutical on a given day for technical quality control.

^99m^Tc-HMPAO and ^99m^Tc-duramycin were prepared as previously described [15,16,18]. Rats were anesthetized (4–5% isoflurane), and a tail vein was cannulated. The rat was then placed supine on a plexiglass plate (4 mm) positioned directly on the face of a parallel-hole collimator (hole diameter = 2 mm, depth = 25 mm) attached to a modular gamma camera (Radiation Sensors, LLC, Harvest, AL, USA) for planar imaging [10,18]. At this point, the concentration of isoflurane was reduced to a level that maintains the proper plane of anesthesia (0.5 to 3%), which was monitored by toe pinch reflex.

An injection of ^99m^Tc-duramycin (37–56 MBq) was administered via the tail vein catheter. ^99m^Tc-duramycin reaches steady-state in the lung by 20 min post-injection, at which time five 30 s planar images were acquired [10,18]. Then, without relocation, an injection of ^99m^Tc-HMPAO (37–56 MBq) was administered via the venous catheter, and images were acquired 20 min post-injection. A final injection of ^99m^Tc-MAA (37 MBq) was made via the same venous cannula, and the rat was reimaged to yield planar images in which the lung boundaries were clearly identified, since >95% of ^99m^Tc-MAA lodges in the lungs [15]. After imaging, the rats recovered and were rehoused.

### 4.5. Image Analysis

The planar images were analyzed using MATLAB-based software (R2025a version, MathWorks, Natick, MA, USA) as described previously [10,18]. For each rat the five images acquired following injection of either ^99m^Tc-duramycin or ^99m^Tc-HMPAO were averaged. Then, the average ^99m^Tc-HMPAO image was subtracted from the average ^99m^Tc-duramycin image to obtain an average ^99m^Tc-HMPAO-only image. The boundaries of the lungs were identified in the high-sensitivity ^99m^Tc-MAA images and manually outlined by a single operator blinded to the treatment group. That full lung region was then truncated by drawing a horizontal boundary at the widest portion of the lung, resulting in a lung ROI free of any liver contribution [10]. The ^99m^Tc-MAA lung ROI mask was then superimposed on the average biomarker image (^99m^Tc-duramycin and ^99m^Tc-HMPAO) yielding a lung biomarker ROI. No registration was required since the animal was maintained in the same location throughout the imaging study. Background regions in the upper forelimbs were also identified in the biomarker image to normalize lung activity for injected biomarker specific activity, dose, and decay [10,18]. Mean counts/pixels within both the lung and forelimb-background ROIs were then determined. The ratio of the lung and background ROI signals was used as the measure of lung biomarker uptake [10,18]. Intra-operator variability of 6% has been previously reported [15].

### 4.6. Pleural Effusion, Lung Wet Weight, and Lung Wet-to-Dry Weight Ratio

At the time of euthanasia (day 120 or when rats met euthanasia criteria), the chest cavity was opened and cotton gauze was inserted to absorb any pleural effusion [14]. The gauze was weighed before and after use, and the difference in weights was reported. Heart and lungs were then isolated as previously described [10]; the lungs were dissected free of the heart, trachea and mainstem bronchi. The right lung lobe was weighed and then dried at 60 °C for 72 h for wet-to-dry weight ratio. Rats that died overnight while not observed did not have their lungs harvested or pleural effusion measured.

### 4.7. Statistical Analysis

Statistical evaluation of data was carried out using GraphPad Prism v. 10.2.3 (Dotmatics Inc., Boston, MA, USA). The level of statistical significance was set at 0.05 for all tests. Group results are expressed as mean ± SEM, unless noted otherwise. Differences in endpoint criteria in Table 1 between the four groups of rats were evaluated using one-way ANOVA with Tukey’s multiple comparison test. Differences in lung uptake of the biomarkers between week 2 and week 4 or differences in mtDAMPs levels between week 2 and week 3 were evaluated using a paired two-tailed *t*-test or Mann–Whitney Rank Sum test as necessary for data that was not normally distributed (Figure 3 and Figure 5). Differences in lung uptake of the biomarkers or mtDAMPS between nonirradiated control and irradiated animals at a given time point were evaluated using an unpaired two-tailed *t*-test, or Mann–Whiteny Rank Sum test (Figure 3 and Figure 5). Differences in the percent change in survivors and nonsurvivors were also evaluated using an unpaired two-tailed *t*-test, or Mann–Whiteny Rank Sum test (Figure 4 and Figure 6).

## 5. Conclusions

Lung uptake of ^99m^Tc-duramycin and ^99m^Tc-HMPAO in irradiated and nonirradiated rats measured relatively early after irradiation (i.e., prior to clinical evidence of radiation pneumonitis) can distinguish subjects likely to go on to recover without treatment from those likely to experience severe pneumonitis. Circulating mtDAMPs offer an additional systemic biomarker associated with outcome and treatment response, with the potential to enhance imaging-based survival prediction and facilitate earlier risk stratification. Together, these findings support an integrated approach for early risk stratification and treatment monitoring after radiation exposure in both countermeasure and clinical radiotherapy contexts [2,3,39].

## Figures and Tables

**Table 1:** Number of days of survival, systemic and lung injury endpoints.

	Nonirradiated	Irradiated	Nonirradiated + Lisinopril	Irradiated + Lisinopril
**Number of days of survival**	120 ± 0 (6)	72.2 ± 6.2 *** (16)	120 ± 0 (4)	105.9 ± 7.1 ^#^ (9)
**Body weight gain, g/day**	0.34 ± 0.02 (6)	−0.11 ± 0.02 *** (16)	0.34 ± 0.02 (4)	0.06 ± 0.04 *** ^# &^ (9)
**Pleural effusion volume, g**	0 ± 0 (6)	6.5 ± 0.9 *** (16)	0 ± 0 (4)	1.1 ± 1.1 ^#^ (7)
**Wet/dry weight**	3.96 ± 0.24 (6)	4.45 ± 0.23 (8)	3.98 ± 0.16 (4)	4.73 ± 0.10 (6)

Values are mean ± SEM. (n) number of rats. Different from nonirradiated: *** *p* < 0.001. Irradiated lisinopril-treated different from irradiated: ^#^ *p* < 0.05. Irradiated lisinopril-treated different from nonirradiated lisinopril-treated: ^&^ *p* < 0.05. (n) is the number of rats.

**Table 2:** Rat classification based on one imaging agent.

Classification	Definition
**True positive (TP)**	[% increase in lung uptake > *Q*)] and [died]
**False positive (FP)**	[% increase in lung uptake > *Q*] and [survived]
**True negative (TN)**	[% increase in lung uptake < *Q*] and [survived]
**False negative (FN)**	[% increase in lung uptake < *Q*] and [died]

**Table 3:** Rat classification based on two imaging agents.

Classification	Definition
TP	[(^99m^Tc-duramycin % increase > *Q_D_*) and (^99m^Tc-HMPAO % increase > *Q_H_*)] and [died]
FP	[(^99m^Tc-duramycin % increase > *Q_D_*) and (^99m^Tc-HMPAO % increase > *Q_H_*)] and [survived]
TN	[(^99m^Tc-duramycin % increase < *Q_D_*) or (^99m^Tc-HMPAO % increase < *Q_H_*)] and [survived]
FN	[(^99m^Tc-duramycin % increase < *Q_D_*) or (^99m^Tc-HMPAO % increase < *Q_H_*)] and [died]

## Data Availability

The original contributions presented in this study are included in the article/Supplementary Materials. The raw data supporting the conclusions of this article will be made available by the corresponding author on request. Further inquiries can be directed to the corresponding author.

## Supplementary Materials

The following supporting information can be downloaded at: https://www.mdpi.com/article/10.3390/ijms27052485/s1.
