# Supplementary material for: Molecular Lung Imaging Following Exposure to Radiation Predicts Long-Term Survival in Rats
Source: Int J Mol Sci. 2026 Mar 8;27(5):2485. doi: 10.3390/ijms27052485 (PMC12986413; doi:10.3390/ijms27052485)
Supplement: Supplementary file 1 [file ijms-27-02485-s001.zip › ijms-4129790-supplementary.pdf]

# Supplemental Materials: *Molecular Lung Imaging Following Exposure to Radiation Predicts Long-term Survival in Rats*

Anne V. Clough[1,2], Kathrina Mpala[3], Pardis Taheri[3], Laura Norwood Toro[7], Andreas M. Beyer[7,8, 9], Tracy Gasperetti[4], Ming Zhao[5], Sarah Kerns[4], Heather A. Himburg[4], and Said H. Audi[2,3,6]

[1]Department of Mathematical and Statistical Sciences, Marquette University
[2]Clement J. Zablocki V.A. Medical Center
[3]Marquette University-Medical College of Wisconsin Department of Biomedical Engineering
[4]Department of Radiation Oncology, Medical College of Wisconsin
[5]Department of Medicine (Cardiology), Feinberg School of Medicine, Northwestern University
[6]Division of Pulmonary and Critical Care Medicine, Medical College of Wisconsin
[7]Department of Medicine, Medical College of Wisconsin
[8]Department of Physiology, Medical College of Wisconsin
[9]A.I.Virtanen Institute for Molecular Sciences University of Eastern Finland, Finland

**Running head:** Molecular Lung Imaging Predicts Rat Survival

**Corresponding Author:**
Said H. Audi, PhD
Research Service 151
Clement J. Zablocki VA Medical Center
5000 W. National Ave
Milwaukee, WI 53295
said.audi@marquette.edu

Table S1 shows the primer sets used for the qRT-PCR analyses targeting four regions of the rat mtDNA in plasma are listed in (Integrated DNA Technologies, Coralville, Iowa).

**Table S1: Primer**

| Primer names | Forward Primer | Reverse Primer | Product Size |
|---|---|---|---|
| ratDloop | CCTCCGTGAAATCAACAACC | TAGTCACCCCCAGGACGAAT | 73 |
| ratATP68 | CCTCTTTCATTACCCCCACA | GGCGTTCTGATGATGGGAAT | 90 |
| ratND45 | CTCACAACACACCCCCTACC | TGCTAGTCATAGAATGGTGTTGGA | 54 |
| ratND12 | CACCCCCTTATCAACCTCAA | AGACTGAAAGACTTGATGTGGCT | 64 |
